# *In situ *protein expression in tumour spheres: development of an immunostaining protocol for confocal microscopy

**DOI:** 10.1186/1471-2407-10-106

**Published:** 2010-03-22

**Authors:** Louis-Bastien Weiswald, Jean-Marc Guinebretière, Sophie Richon, Dominique Bellet, Bruno Saubaméa, Virginie Dangles-Marie

**Affiliations:** 1 IFR71 Sciences du Médicament, Faculté des Sciences Pharmaceutiques et Biologiques, Université Paris Descartes, Paris, France; 2 Service de Pathologie, Hôpital René Huguenin, Institut Curie, Saint Cloud, France; 3 Service de Médecine Nucléaire, Laboratoire d'Oncobiologie, Hôpital René Huguenin, Institut Curie, Saint Cloud, France; 4 INSERM U705, UMR CNRS 8206 and Plateau Technique d'Imagerie Cellulaire et Moléculaire, IFR71 Sciences du Médicament, Faculté des Sciences Pharmaceutiques et Biologiques, Université Paris Descartes, Paris, France; 5 Current address: Preclinical Investigation Laboratory, Department of Translational Research, Institut Curie, INSERM U932, Université Paris Descartes, Paris, France

## Abstract

**Background:**

Multicellular tumour sphere models have been shown to closely mimic phenotype characteristics of *in vivo *solid tumours, or to allow *in vitro *propagation of cancer stem cells (CSCs). CSCs are usually characterized by the expression of specific membrane markers using flow cytometry (FC) after enzymatic dissociation. Consequently, the spatial location of positive cells within spheres is not documented. Confocal microscopy is the best technique for the imaging of thick biological specimens after multi-labelling but suffers from poor antibody penetration. Thus, we describe here a new protocol for *in situ *confocal imaging of protein expression in intact spheroids.

**Methods:**

Protein expression in whole spheroids (150 μm in diameter) from two human colon cancer cell lines, HT29 and CT320X6, has been investigated with confocal immunostaining, then compared with profiles obtained through paraffin immunohistochemistry (pIHC) and FC. Target antigens, relevant for colon cancer and with different expression patterns, have been studied.

**Results:**

We first demonstrate that our procedure overcomes the well-known problem of antibody penetration in compact structures by performing immunostaining of EpCAM, a membrane protein expressed by all cells within our spheroids. EpCAM expression is detected in all cells, even the deepest ones. Likewise, antibody access is confirmed with CK20 and CD44 immunostaining. Confocal imaging shows that 100% of cells express β-catenin, mainly present in the plasma membrane with also cytoplasmic and nuclear staining, in agreement with FC and pIHC data. pIHC and confocal imaging show similar CA 19-9 cytoplasmic and membranar expression profile in a cell subpopulation. CA 19-9^+ ^cell count confirms confocal imaging as a highly sensitive method (75%, 62% and 51%, for FC, confocal imaging and pIHC, respectively). Finally, confocal imaging reveals that the weak expression of CD133, a putative colon CSC marker, is restricted to the luminal cell surface of colorectal cancer acini, with CD133^+ ^cellular debris into glandular lumina.

**Conclusion:**

The present protocol enables *in situ *visualization of protein expression in compact three-dimensional models by whole mount confocal imaging, allowing the accurate localization and quantification of cells expressing specific markers. It should prove useful to study rare events like CSCs within tumour spheres.

## Background

In a constant effort to produce more and more pertinent *in vitro *models for cancer studies, the importance of studying cancer cells in three-dimensions (3D) is increasingly recognized [1-5]. For this purpose, spheroids can be produced *in vitro *with some permanent cancer cell lines, including colon cancer cell lines, when cultured in non-adherent conditions [6,7]. These spheroids are known to mimic microtumours more closely than cancer cell line monolayers and have been mainly used in chemo- and radio-resistance studies. Indeed, tumour spheroids represent quite realistically the three-dimensional growth and organization of solid avascular tumours and, consequently, simulate much more precisely the cell-cell interactions and microenvironmental conditions found in tumours, especially nutrient and oxygen gradients. Another 3D cancer cell model, requiring a specific culture protocol, aims to promoting *in vitro *expansion of cancer stem cells (CSCs) from solid tumour tissue as is the case with neurospheres [8,9], mammospheres [10,11] or colon cancer spheres [12,13]. CSCs are defined as a rare subset of tumour cells, which have the unique capability to form tumours in serial xenotransplantation assays, and to reestablish, at each *in vivo *passage, the hierarchical cell organization and heterogeneity of the parental tumour. *In vitro *methods have been developed to grow and study these cells in sphere-forming assays. The phenotypic cell isolation strategy that relies on the immunotargeting of cell surface proteins coupled with cell sorting by flow cytometry (FC) is now widely used to isolate CSCs, after enzymatic dissociation of tumour samples or cancer spheres.

Up to now, protein expression by these sphere forming cells was mostly studied by FC on single cells. Immunostaining techniques performed on the whole intact spheres are traditionally done on cytospin preparations [14,15] and paraffin [16,17] or frozen sections [13,17] because of the common failure of the antibodies to penetrate fully into compact tumour spheres, leading to a loss of information. Besides, the cytospin approach is limited to the investigation of the more external cells, while physical sections do not allow the analysis of the entire 3D structure, unless time-consuming serial sectioning is performed. Another way to gain access to the whole structure has been to use confocal imaging of three dimensional tumour spheroids expressing genetically encoded fluorescent reporters fused to the proteins of interest [18,19]. However, this approach requires a cell transfection step and does not allow visualizing endogenous proteins at their physiological levels.

In the present work, we describe a new immunostaining protocol in which spheroids are simultaneously fixed and permeabilized in a mixture of paraformaldehyde and Triton X100 and then gradually dehydrated in methanol before incubation with labelling antibodies. We show that this results in the complete permeabilization of the spheroids while preserving their structures and the localization of the proteins of interest. Whole spheroids can then be imaged in the confocal microscope to reveal the spatial distribution patterns of several endogenous proteins *in situ*.

## Methods

### Cell culture

CT320X6, established in the laboratory [20], and HT29 (ATCC, HTB-38) colon cancer cell lines were maintained in DMEM supplemented with 10% FCS, 10 mmol/L HEPES, 4.5 g/L glucose, 1 mol/L pyruvate sodium, 200 units/mL penicillin, 200 μg/mL streptomycin at 37°C, with 8% CO_2_.

Three-dimensional multicellular spheroids were prepared by the liquid overlay technique [6]. In brief, tissue culture microplates were coated with 75 μl of 1% agarose in water. CT320X6 and HT29 cells grown as a two-dimensional monolayer were resuspended with trypsin, and 2 × 10^3 ^(CT320X6) or 1 × 10^3 ^(HT29) cells were seeded in 150 μl of culture medium to obtain a single spheroid per microwell with a diameter between 120 and 150 μm after 4 days.

### Antibodies

Primary antibodies used for flow cytometry, paraffin immunohistochemistry and confocal microscopy are recapitulated in Table 1. AlexaFluor^®^488 conjugated goat anti-mouse and AlexaFluor^®^555 conjugated goat anti-rabbit secondary antibodies were from Invitrogen (Cergy Pontoise, France). The results shown in all figures are from one experiment taken as representative of at least three independent experiments.

**Table 1:** Primary antibodies used in the study.

				Antibody dilution		
				
Targeted antigen	Clone name	Manufacturer	Ab type	^*a*^FC	^*b*^pIHC	Confocal imaging
***Primary antibodies***						
Ep-CAM	HEA-125	Miltenyi-Biotec SAS	FITC-mouse IgG1	1:11	-	1:50
CK20	IT-Ks 20.10	Biovalley	FITC-mouse IgG1	1:50	-	1:50
Beta-Catenin	β-catenin-1	DAKO	mouse IgG1	1:20	-	1:50
Beta-Catenin	14/Beta-Catenin	BD Biosciences	mouse IgG1	-	1:400	-
CA 19-9	C241:5:4:1	Novacastra	mouse IgG1	1:20	1:200	1:50
CD133	AC133	Miltenyi-Biotec SAS	mouse IgG1	1:11	-	1:50
EBP50	-	Calbiochem	rabbit^*c*^pAb	-	-	1:300
CD44	G44-26	BD Biosciences	mouse IgG2b	1:50	1:10	1:50
ALDH1	44/ALDH	BD Biosciences	mouse IgG1	-	1:100	1:100

^*a*^FC: flow cytometry; ^*b*^pIHC: paraffin immunochemistry; ^*c*^pAb: polyclonal antibody

### Flow cytometry analysis

Spheroids were disaggregated with trypsin (0.025%)/EDTA (2 mmol/L) and cell concentration was adjusted to 1 × 10^6 ^cells/ml with DMEM supplemented with 10 mmol/L HEPES, 4.5 g/L glucose, 1 mmol/L pyruvate sodium.

For EpCAM, CD44 and CD133 staining, living cells were incubated for 30 minutes at 4°C with primary antibody and eventually followed by anti-mouse AlexaFluor^®^488 secondary antibody (30 min at 4°C). CK20, β-catenin and CA 19-9 staining was performed after cell fixation and permeabilization using Intrastain kit (DAKO, Trappes, France) according to the instructions of the manufacturer.

Labelled cells were analyzed by an Epics XL cytometer (Coulter, Villepinte, France) and data were computed using WinMDI 2.9 software (Joseph Trotter, Scripps Research Institute, La Jolla, CA, USA). Gating was done on the basis of negative-control staining profiles, obtained by substituting primary antibodies with isotypic non immune IgGs.

### Paraffin immunohistochemistry

Spheroids were embedded using the Cytoblock™ method and the Shandon kit (Thermo electroncorporation, Saint Herblay, France) [21]. Immunostaining was performed using an automated immunostainer (Dako Autostainer, Dako) with appropriate antibody dilution (see Table 1). Dako REAL™ system (Dako) was used for antibody binding detection according to the manufacturer's instructions.

### Confocal microscopy

#### Fixation/permeabilization

About 100 spheroids in suspension were fixed and permeabilized for 3 h at 4°C in phosphate buffered saline (PBS) containing 4% PFA (Euromedex, Mundolsheim, France) and 1% Triton X-100 (Perbio Science, Brébières, France) and washed in PBS (3 × 10 min). Spheroids were then dehydrated in an ascending series of methanol at 4°C in PBS (25%, 50%, 75%, 95%, 30 min each and 100% for 5 h), rehydrated in the same descending series and washed in PBS (3 × 10 min).

#### Antibody staining

After blocking in PBST (0.1% Triton X-100 in PBS) containing 3% Bovine Serum Albumin (MP Biomedicals, Illkirch, France) overnight at 4°C and washing in PBST (2 × 15 min), spheroids were incubated with primary antibodies diluted in PBST on a gently rocking rotator at 4°C for 48 h and rinsed in PBST (4 × 30 min). When necessary, spheroids were then incubated in appropriate AlexaFluor conjugated secondary antibodies for 24 h. Cell nuclei were eventually counterstained by TOPRO-3 (Invitrogen) diluted 1:500 in PBS for 40 min at room temperature.

#### Mounting

Mounting was carried out in a simple chamber assembled from a glass slide and a cover slip, using double-sided scotch as 'spacer' between them. Spheroids were resuspended in 15 μl of PBS and allowed to adhere on a SuperFrost glass slide (Roth, Karlsruhe, Germany). After careful blotting of excess buffer, spheroids were mounted in 15 μl of 90% glycerol (v/v in PBS) using 0.17 mm thick coverslips (Assistent, Sondheim, Germany). Edges of the coverslip were sealed with nail polish.

#### Confocal image acquisition and analysis

Images were recorded on a Leica TCS SP2 confocal microscope (Leica Microsystems, Wetzlar, Germany) equipped with a 40× oil-immersion objective (NA = 1.32). The three channels were acquired sequentially with the following excitation and emission parameters: (488 nm, 500-540 nm) for Alexa 488 and FITC, (543 nm, 555-615 nm) for Alexa 555 and (633 nm, 645-750 nm) for TOPRO-3. Gains were adjusted to avoid saturation in pixels intensity. The three channels were merged using Image J software.

Negative controls, in which primary antibodies were substituted with isotypic non immune IgGs, did not give rise to any detectable labelling. No post-imaging treatment was used except for TOPRO-3 images which were systematically despeckled in Image J.

CA 19-9 and ALDH1 positive and negative cells within a spheroid were manually counted using the Cell Counter plug-in of the ImageJ software and the percentage of positive cells was determined at a depth of 40 μm and 70 μm relative to the coverslip. No significant difference was observed between both depths within the same spheroid. This analysis was performed for three independent experiments from which mean and SD values were determined.

#### 3D reconstruction

For 3D reconstruction, a stack of confocal images was collected through the spheroids with step size of 0.488 μm between adjacent optical planes, starting from one pole of the spheroids. After thresholding, this stack was used to generate a 3D animation sequence by using the 3D Projection routine in Image J.

### Statistical analysis

Percentage of positive cells detected by the different techniques was compared pairwise using a Wilcoxon-Mann-Whitney one-sided test. A *p *value of less than 0.05 was considered to be significant.

## Results and discussion

The method presented here aims to visualizing *in situ *the expression of proteins in three-dimensional tumour models using immunostaining coupled with confocal imaging. We used HT29 and CT320X6 colon cancer cell lines because of their ability to form compact multicellular tumour spheroids when cultured according to the liquid overlay technique [6]. All spheroids used had a diameter comprised between 120 and 150 μm.

**Figure 1:**
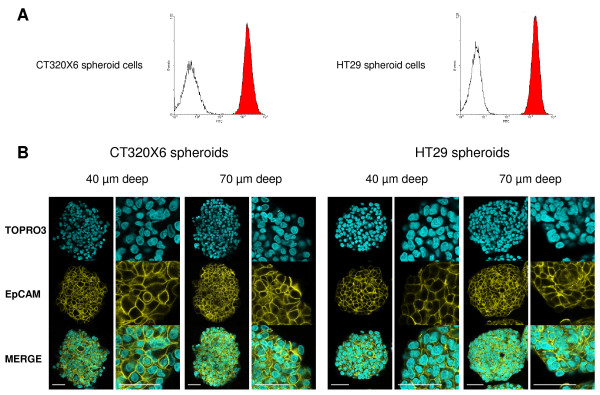
**Homogeneous penetration of EpCAM-FITC antibody in whole spheroids**. (A) Flow cytometry analysis of CT320X6 (left histogram) and HT29 (right histogram) cells from dissociated spheroids. Cells were labelled with the same FITC-conjugated EpCAM antibody (red area) or FITC-conjugated isotype control antibody (white area). All cells were EpCAM positive. (B) CT320X6 (left panels) and HT29 (right panels) spheroids were fixed and permeabilized as described in *Methods*. Spheroids were then directly labelled using FITC-conjugated EpCAM antibody (yellow) and counterstained with TOPRO-3 (cyan). Acquisitions were taken at 40 μm and 70 μm depth within the same spheroid (scale bar = 50 μm).

One of the main problems arising in confocal imaging of thick specimens is the poor penetration of the antibodies within the whole structure. Therefore, we first addressed this issue by performing confocal imaging after immunostaining of the spheroids against a marker expressed evenly by all cells within the spheroids. We chose Epithelial Cell Adhesion Molecule (EpCAM), a membrane protein also known as Epithelial-Specific Antigen (ESA) since all cells of the HT29 and CT320X6 spheroids are known to be positive for EpCAM, as confirmed by FC analysis (Figure 1A). After immunostaining with an anti-EpCAM monoclonal antibody conjugated to FITC, confocal images were recorded in single optical planes located 40 μm and 70 μm below the surface of the coverslip.

As explained below, the main problem we had to deal with was the penetration of the antibody through the whole spheroid. The fixation and permeabilization steps appeared to be the most critical ones to achieve this goal.

A classical two steps method, namely fixation in 4% PFA (3 h, 4°C) followed by permeabilization in 1% Triton X100 (1 h, RT) did not yield satisfactory results. This procedure resulted in a bright anti-EpCAM staining of cell plasma membranes in the periphery of the spheroid but left the centre of the spheroid without any detectable labelling (Additional file 1A). This was in contrast to staining with TOPRO-3, a small cell-impermeant molecule, which stained all cell nuclei in the spheroids. Changing the experimental conditions for the fixation (2-4% PFA, 1-12 h, RT or 4°C) or the permeabilization (0.5-3% Triton X100, 1-6 h, RT or 4°C), using other detergents (saponine, SDS) or increasing incubation times in antibodies (up to 72 h) did not lead to any improvement (data not shown). The reverse protocol consisting in extraction (0.1% Triton X100, 5 min, 4°C) prior to fixation (4% PFA, 3 h, 4°C) resulted in cell membrane damage in the periphery of the spheroids with seemingly no permeabilization in the centre (Additional file 1B). Simultaneous fixation/permeabilization in a single step led to a striking increase of the staining inside the spheroids with no apparent alteration of the structure (Additional file 1C). Indeed, when spheroids were incubated in PBS containing 4% PFA and 1% Triton X-100 at 4°C for 3 h before immunostaining, all cells were evenly labelled down to a depth of 40 μm. However, the very center of the spheroid still appeared devoided of any staining as evidenced in images taken at 70 μm depth (Additional file 1D).

Dehydration in alcohols has been reported to efficiently permeabilize thick specimens, thus allowing full penetration of antibodies or fluorescent probes. For example, dehydration in methanol followed by rehydration allowed whole-mount immunostaining of large insect brains [22] or microvasculature imaging in whole-mount organs [23]. Moreover, only minimal, and more importantly isotropic, shrinkage occurs when alcohol concentration is gradually increased [24]. After simultaneous fixation/permeabilization as described above we then passed the spheroids through a series of baths of increasing methanol concentration at 4°C (see Methods). The spheroids were then rehydrated through the same descending series of baths and resuspended in PBS before immunostaining.

As can be seen in Figure 1B, this procedure resulted in a strong and uniform membrane staining with an anti-EpCAM antibody in both HT29 and CT320X6 spheroids. The same staining pattern was observed at 40 μm and 70 μm depth, thus demonstrating the full penetration of the antibody down to the center of the spheroids. The dramatic increase in permeability observed after methanol treatment likely results from two effects. Indeed, methanol is both a strong permeabilizing agent which 'dissolves' the membranes more efficiently than nonionic detergents like Triton X100 and a coagulative fixative [25] which probably makes the cytosol matrix less gelatinous and thus more permeable to antibodies. However, a simplified procedure consisting in a single step of fixation/permeabilization in cold methanol or methanol/acetone without prior aldehydic fixation led to a massive delocalization of membrane proteins (data not shown). This is in contrast with a previous work reporting the successful fixation and permeabilization of embryoid bodies by cold methanol/acetone treatment without observation of membrane damage [26]. Antigen delocalization is a known possible artefact in immunostaining experiments [27,28]. It can occur during the fixation and/or permeabilization steps and lead to a wrong conclusion regarding the subcellular localization of the labelled proteins. In the current study, we found that aldehydic fixation before methanol treatment allowed EpCAM to be correctly localized at the plasma membrane. This was also the case for all antigens examined (see below) whatever the subcellular location (cytosolic, nuclear, cytoskeleton- and membrane-associated proteins). Nevertheless the possibility of this artefact should be kept in mind when addressing a new target since it is dependent of both the tissue and the antigen examined. It is also noteworthy that, although the present protocol implies long processing times, it can be performed in several steps. In particular, after dehydration/rehydration in methanol, spheroids could be stored for up to one month at 4°C in PBS without any alteration in the staining pattern of the antigens examined. This protocol was used in all subsequent immunostaining experiments.

To further validate our protocol, *in situ *expression of other proteins was studied and compared to the expression profiles obtained by FC or paraffin immunochemistry (pIHC). We selected several antigens for their different subcellular distribution (cytoplasm and/or nucleus and/or plasma membrane) and their relevance in the field of colon cancer.

**Figure 2:**
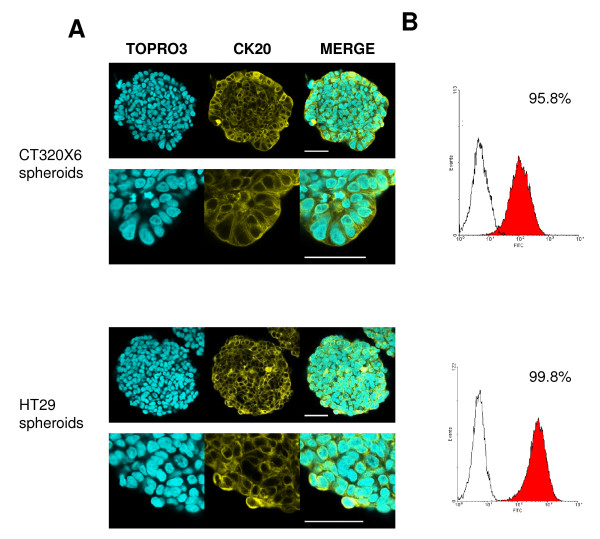
**CK20 expression in colon cancer spheroids**. (A) CT320X6 (upper panel) and HT29 (lower panel) spheroids were immunostained against CK20 (yellow) and nuclei were counterstained with TOPRO-3 (cyan). Acquisitions were taken at 70 μm depth. Almost all cells were positive (scale bar = 50 μm). (B) Permeabilized cells from dissociated CT320X6 (upper panel) and HT29 (lower panel) spheroids were labelled with the same mAb against CK20. FC analysis confirmed a population with almost 100% of positive cells.

Cytokeratin 20 (CK20) is an intermediate filament protein whose presence is essentially restricted to differentiated cells from gastric and intestinal epithelium and urothelium [29]. CK20 is routinely used in combination with CK7 in distinguishing colon carcinomas (CK20^+^) from ovarian, pulmonary and breast carcinoma [30]. Both HT29 and CT320X6 spheroids are formed with well-differentiated human colon adenocarcinoma cell lines and cultured in serum-containing medium. The CK20 expression profile observed here is consequently in agreement with these data. Indeed, almost all cells in HT29 and CT320X6 spheroids were found positive in confocal imaging and also FC analysis (Figure 2).

**Figure 3:**
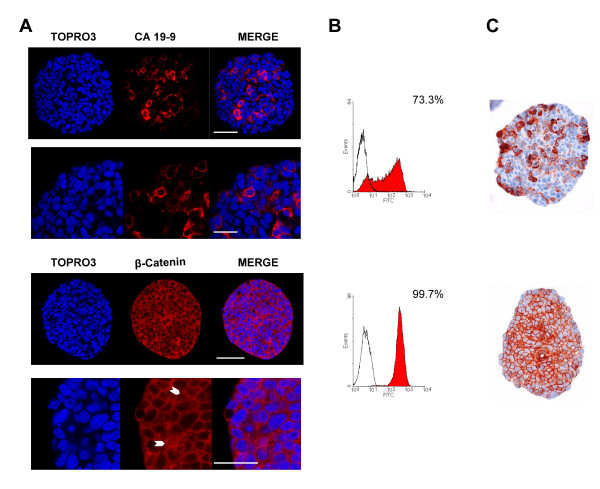
**CA 19-9 and β-catenin expression in HT29 spheroids**. (A) HT29 spheroids were immunostained against CA 19-9 (red, upper panel) or β-catenin (red, lower panel) and nuclei were counterstained with TOPRO-3 (blue). Acquisitions were taken at 70 μm depth. HT29 spheroids displayed a heterogeneous CA 19-9 expression with a subpopulation of 28% of CA 19-9 negative cells while cytoplasmic, nuclear and membrane associated β-catenin was found in virtually all cells. White arrowheads show nuclear β-catenin immunostain. Scale bar = 50 μm (whole spheroid) and 25 μm (zooms). (B) Permeabilized cells from dissociated HT29 spheroids were labelled against CA 19-9 (upper histogram) or β-catenin (lower histogram) and analysed by flow cytometry. All HT29 spheroid cells were positive for β-catenin whereas 26% of cells were negative for CA 19-9. (C) HT29 spheroids were embedded using the Cytoblock method and stained with the anti-CA 19-9 antibody (upper image) or the anti-β-catenin antibody (lower image) using conventional immunohistochemistry and counterstained with hematoxilin. A subset of cells did not express CA 19-9 (49%) while β-catenin staining was observed in nearly all cells, mainly associated with plasma membrane but also within cytoplasm and nuclei.

The cytosolic carbohydrate antigen CA 19-9 is a glycoprotein related to the monosyalilated Lewis antigen, which is expressed in the cell membrane and the cytosol of human colorectal carcinoma as well as in the normal mucosa [31]. CA 19-9 is frequently used as a tumour marker, particularly for colon and pancreas cancer and CA 19-9 serum level is considered a reliable indicator for patient follow-up [32]. The three immunostaining techniques revealed both CA 19-9 positive and negative cell populations within HT29 spheroids (Figure 3). However, these techniques were not quantitatively equivalent even when the same antibody was used. Unsurprisingly, FC was the most sensitive technique with 75 ± 2.6% CA 19-9 positive cells detected while confocal microscopy (62 ± 6.1%) turned out to have a higher sensitivity than pIHC (51 ± 0.6%, p < 0.05). Besides, the location pattern of CA 19-9 was qualitatively similar in confocal imaging (Figure 3A) and in pIHC (Figure 3C), thus confirming the validity of our protocol for this marker.

β-catenin plays a dual role in cells as a structural component of cell-cell adherens junctions and as a key player in the Wnt signalling pathway [33]. This pivotal protein thus displays a complex localization pattern, being present at the plasma membrane (associated with E-cadherin), the cytosol (within a multiprotein complex containing notably GSK3β, axin, and APC) and the nucleus (where it acts as a co-transcriptional activator of target genes and promotes tumour progression). The nuclear accumulation of the β-catenin is considered the initial step in colon carcinogenesis and the transcription of β-catenin target genes becomes constitutively activated in APC-deficient colon cancers [34]. However, most tumours do not display a homogeneous subcellular expression pattern but a nuclear accumulation of β-catenin predominantly localized at the tumour's periphery [35]. All cells from HT29, an APC-deficient cell line, were positive for β-catenin, as illustrated by FC analysis (Figure 3B). This was confirmed by confocal microscopy (Figure 3A) which gave additional information about the subcellular distribution of the protein. Besides being strongly expressed at the level of the plasma membrane, soluble β-catenin was often found in cytosolic and nuclear compartments in confocal images, as confirmed by pIHC (Figure 3C). This nuclear expression was randomly detected within the spheroids. More recently, nuclear β-catenin has been also presumed to mark colon cancer stem cells [13,36,37].

Because they are suspected to play a crucial role in the initial steps of tumorigenesis, cancer stem cells are the subject of intensive investigations. Floating multicellular spheres (including colon cancer spheres, mammospheres or neurospheres) have been described as being valuable models for *in vitro *CSC expansion and are topologically similar to the spheroids used in the present study. The expression of specific CSC markers within these microspheres is traditionally investigated by FC after enzymatic dissociation. This is a powerful approach allowing to rapidly and quantitatively assess the presence of CSCs in the spheres and also to isolate CSCs which can then be tested for *in vivo *tumourigenicity. However, it can not address the precise distribution of CSC markers within the spheres, for example in clustered or isolated cells.

**Figure 4:**
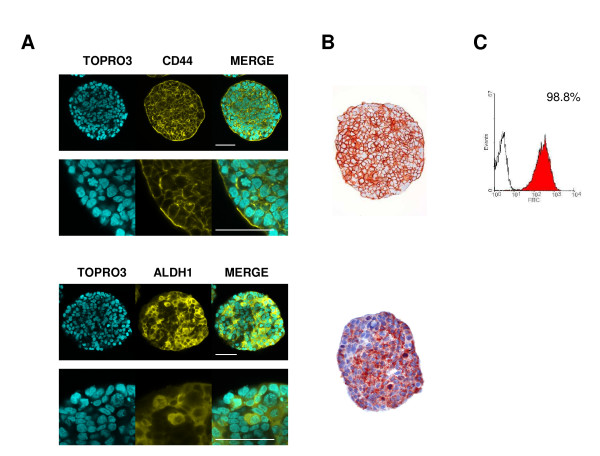
**CD44 and ALDH1 expression in HT29 spheroids**. (A) HT29 spheroids were immunostained against CD44 (yellow, upper panel) or ALDH1 (yellow, lower panel) and nuclei were counterstained with TOPRO-3 (cyan). Acquisitions were taken at 70 μm depth (scale bar = 50 μm). All cells expressed CD44 while a large population of cells (86%) displayed ALDH1 staining. (B) Conventional pIHC using Cytoblock embedding technique confirmed that all cells were CD44^+ ^and 85% were ALDH1^+^. (C) All cells from dissociated HT29 spheroids were found CD44^+ ^in FC analysis.

We applied the method described in the present work to study the localization of putative CSC marker-expressing cells within HT29 and CT320X6 spheroids. It has to be noted that the liquid overlay technique in conventional culture medium used here is not a method dedicated to CSC expansion. However, this technique allows the obtaining of reproducible and compact cancer cell spheroids, mimicking strong cell-cell interactions occurring in CSC spheres. First, CD44, a widely distributed transmembrane cell adhesion molecule and the major cell surface receptor for hyaluronic acid, is reported for isolating colorectal cancer initiating cells [38-40]. Confocal imaging, FC and pIHC studies revealed that all cells within HT29 spheroids displayed CD44 expression (Figure 4). These results are in line with previous phenotyping of HT29 cells [41]. ALDH1, largely reported as breast CSC marker [42,43], is also a potential stem cell marker for colon cancer [44,45]. In the present study, a large population of ALDH1^+ ^cells has been detected within HT29 spheroids both in confocal imaging (86% ± 2) and with pIHC (85% ± 3) (Figure 4). These data are consistent with strong ALDH1 protein staining observed in patient specimens of colon adenocarcinoma, while only few cells were found in FC assays to display ALDH enzymatic activity, a potential characteristic of CSCs [44].

**Figure 5:**
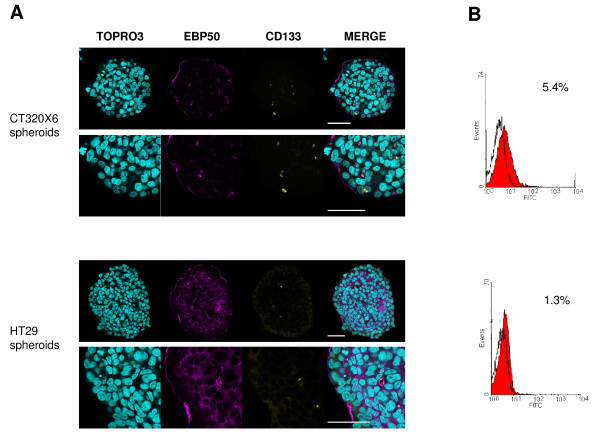
**CD133 expression at the luminal surface of some epithelial tumour glands with shedding into the lumina within tumour spheroids**. (A) CD133 (yellow) and EBP50 (magenta) were visualized within CT320X6 (upper panel) and HT29 (lower panel) spheroids by immunofluorescence staining. Spheroids were counterstained with TOPRO-3 (cyan). CD133 was found into the lumina and colocalized with EBP50 at the luminal surface of some acini. Acquisitions were taken at 70 μm depth (scale bar = 50 μm). (B) Dissociated cells from CT320X6 (upper histogram) and HT29 (lower histogram) spheroids were similarly labelled against CD133 and analysed by flow cytometry.

CD133 (prominin-1) is a five-transmembrane domain glycoprotein localized in membrane protrusions. It is suspected to be a CSC marker in brain [9,46] or colon [12,47] tumours, although this view has been challenged [48,49]. FC analysis (Figure 5B) showed that about 5.0% ± 0.4 of the cells were CD133 positive in CT320X6 spheroids. This percentage was close to 1.1% (n = 2) in HT29 spheroids, a value consistent with a previous report in HT29 cells [50]. Confocal microscopy also identified a small fraction of CD133 positive cells in CT320X6 and HT29 spheroids (Figure 5A) and double staining experiments additionally revealed that CD133 was colocalized with EBP50, a protein found at the apical membrane of polarized epithelia and involved in microvilli formation [51]. At high magnification, EBP50 staining also revealed the presence of glandular acini filled with CD133 positive material. Interestingly, a similar observation was made recently in human colorectal cancer where CD133 was found at the glandular-luminal surface of cancerous cells and in shed cellular debris inside the glandular lumina [52]. Our method is therefore able to reveal fine features in the distribution of specific markers, even with a very low expression, throughout the entire spheroids.

## Conclusion

Different methods have been reported to study protein expression within tumour spheres. Immunostaining detected by peroxidase or fluorophore-conjugated antibodies has been traditionally performed using cytospin preparations [14,15], paraffin sections [16,17] or frozen sections [13,17]. Nevertheless, cytospin slides allow study of only external cells of the structure while paraffin and frozen sections do not permit *in situ *visualization of the target protein in the entire structure.

We have developed a protocol allowing successful antibody penetration in tumour spheres of 150 μm in diameter with excellent preservation of the structure and without artefactual delocalization of the proteins examined. This allowed us to perform whole mount immunostaining of these compact structures and to use the optical sectioning capability of confocal microscopy to analyse the distribution of several proteins of interest in the entire spheroids. Besides, 3D localization of the antigens of interest can be easily obtained from a z-series of confocal images collected through the spheroid (see Additional File 2). Our method is simple, has a high sensitivity and offers the possibility to screen the whole structure. It also makes multi-staining and colocalization of several antigens possible. Therefore, it should prove useful as a new tool to image the subcellular distribution of relevant markers or to localize rare positive cells, like CSCs, within tumour spheres.

## Competing interests

The authors declare that they have no competing interests.

## Authors' contributions

LBW contributed to the conception of the method, performed all confocal experiments in the laboratory, and co-drafted the manuscript. JMG and SR participated in method development, performed paraffin immunostaining and cytometry analysis respectively, and revised the manuscript. DB contributed with data analysis and participated in revision of the manuscript. BS participated in method development, contributed with data analysis and revision of the manuscript. VDM initiated the project, contributed with data analysis and co-drafted the manuscript. All authors read and approved the final manuscript.

## Pre-publication history

The pre-publication history for this paper can be accessed here:

http://www.biomedcentral.com/1471-2407/10/106/prepub

## Supplementary Material

Additional file 1 **Unsuccessful protocols for whole-mount staining of tumour spheroids**. CT320X6 spheroids were labelled using a FITC-conjugated EpCAM antibody (yellow) and counterstained with TOPRO-3 (cyan) after fixation/permeabilization according to one of the protocols described below. Images were recorded at a depth of 40 μm (A, B, C) or 70 μm (D) relative to the coverslip. (A) Fixation in PFA 4% (3 h at 4°C) followed by permeabilization in Triton X-100 1% (1 h at RT). Antibody penetration was limited to the first layer of cells (B) Extraction in Triton X-100 0.1% (5 min at 4°C) followed by fixation in PFA 4% (3 h at 4°C). Antibody penetrated poorly in the center of the spheroids while cell membranes were damaged at the periphery. (C-D) Simultaneous fixation/permeabilization in PFA 4% and Triton X-100 1% (3 h at 4°C). Acquisition at 40 μm (C) yielded a section with homogeneous staining and good preservation of the membranes but optical sections at 70 μm depth (D) showed a poor penetration of the Ab in the center of the spheroids. Click here for file

Additional file 2 **3D reconstruction from a z-series of confocal images taken through a spheroid grown from HT29 cells and immunostained against CA 19-9 (red)**. Nuclei are counterstained with TOPRO-3 (blue). Only a half of the spheroid is presented for better clarity. Click here for file
