# Supplementary figures and images for: In situ protein expression in tumour spheres: development of an immunostaining protocol for confocal microscopy
Source: BMC Cancer. 2010 Mar 22;10:106. doi: 10.1186/1471-2407-10-106 (PMC2851689; doi:10.1186/1471-2407-10-106)

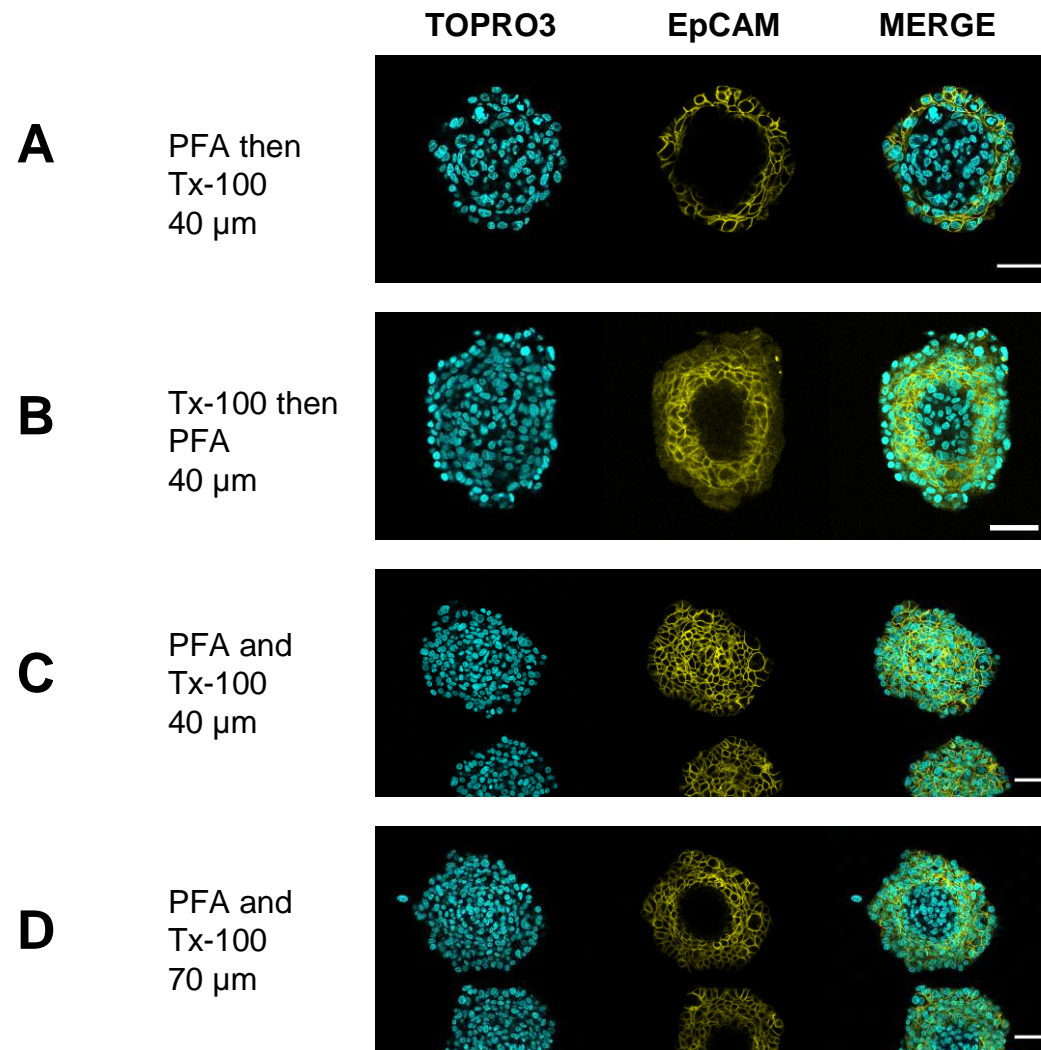

Supplement: Additional file 1 — Unsuccessful protocols for whole-mount staining of tumour spheroids. CT320X6 spheroids were labelled using a FITC-conjugated EpCAM antibody (yellow) and counterstained with TOPRO-3 (cyan) after fixation/permeabilization according to one of the protocols described below. Images were recorded at a depth of 40 μm (A, B, C) or 70 μm (D) relative to the coverslip. (A) Fixation in PFA 4% (3 h at 4°C) followed by permeabilization in Triton X-100 1% (1 h at RT). Antibody penetration was limited to the first layer of cells (B) Extraction in Triton X-100 0.1% (5 min at 4°C) followed by fixation in PFA 4% (3 h at 4°C). Antibody penetrated poorly in the center of the spheroids while cell membranes were damaged at the periphery. (C-D) Simultaneous fixation/permeabilization in PFA 4% and Triton X-100 1% (3 h at 4°C). Acquisition at 40 μm (C) yielded a section with homogeneous staining and good preservation of the membranes but optical sections at 70 μm depth (D) showed a poor penetration of the Ab in the center of the spheroids. [file 1471-2407-10-106-S1.PDF]
